# RNAi-Mediated Knockdown of Serine Protease Inhibitor Genes Increases the Mortality of *Plutella xylostella* Challenged by Destruxin A

**DOI:** 10.1371/journal.pone.0097863

**Published:** 2014-05-16

**Authors:** Pengfei Han, Jiqiao Fan, Yu Liu, Andrew G. S. Cuthbertson, Shaoqiao Yan, Bao-Li Qiu, Shunxiang Ren

**Affiliations:** 1 Engineering Research Center of Biological Control, Ministry of Education, South China Agricultural University, Guangzhou, China; 2 The Food and Environment Research Agency, Sand Hutton, United Kingdom; Ghent University, Belgium

## Abstract

Destruxin A is a mycotoxin that is secreted by entomopathogenic fungi which has a broad-spectrum insecticidal effect. Previous transcript and protein profiling analysis showed that destruxin A has significant effects on the expression of serine protease inhibitor genes (serpin-2, 4, 5) in the larvae of *Plutella xylostella*. In the current study, we aimed to understand the role of serpins under application of destruxin A. We obtained two full-length cDNA sequences of *P*. *xylostella* serpins, named serpin-4 and serpin-5, and cloned the serpin-2 gene whose full-length has already been published. Phylogenetic analysis indicated that these two serpin genes were highly clustered with other serpins associated with the immune response in other insects. The temporal and spatial expression of serpin-2, serpin-4 and serpin-5 were determined to be the highest in the fat body and hemolymph of 4^th^ larval stage using qRT-PCR and western blot detection techniques. RNA interference (RNAi) mediated knockdown of *P*. *xylostella* serpin genes was carried out by microinjection of double-stranded RNA (dsRNA). The expression levels of serpins decreased significantly after RNAi. Results showed that the depletion of serpins induced cecropins expression, increased phenoloxidase (PO) activity, body melanization and mortality in the larvae of *P. xylostella* under the same lethal concentration of destruxin A. The superimposed effects of serpins RNAi were similar with the destruxin A treatment upon mortality of *P*. *xylostella* larvae. We discovered for the first time that serpins play indispensable role in *P*. *xylostella* when challenged by destruxin A and deduced the possible function mechanism of destruxin A. Our findings are conducive to fully understanding the potential insecticidal mechanism of destruxin A and constitute a well-defined potential molecular target for novel insecticides.

## Introduction

Like many invertebrates, insects have a potent and efficient innate immune system. This is considered to constitute an evolutionarily defense strategy, including cellular and humoral immunity that protects the host from infection by other organisms in a non-specific manner [1]. The innate immune processes including body melanization, blood coagulation, cell encapsulation, phagocytosis, complement activation, and synthesis of antimicrobial peptides are regulated by the protease cascades resulting in multiple steps of protease activation [2]–[4]. Lots of serine protease inhibitors (Serpins) play important roles in modulating several immune processes by inactivating the excessive protease activities [5]–[7].

Serpins are a superfamily of proteins that perform a broad spectrum of different biological functions. They are extensively dispersed in many organisms including viruses, bacteria, fungi, plants and animals [8], [9]. Serpins consist of a single chain generally that include 350–450 amino acid residues and form a conserved structure with a reactive center loop (RCL) near the C-terminus, which acts as a binding site for a target protease [10]. The inhibition of proteolysis occurs by forming irreversible 1∶1 complexes between serpins and their target peptidases [11]. Several intracellular as well as extracellular serpins have been identified in the insect genome [12]–[14], with most of these being reported to participate in the regulation of innate immune responses such as modulating prophenoloxidase (pro-PO) activation, hemolymph coagulation and synthesis of antimicrobial peptides (AMPs) [15]. AMPs are important effectors of innate immune response. Cecropins belong to the AMPs family and are synthesized in response to invaders in humoral immune response [16]. In *Drosophila*, serpin43Ac influences the expression of antimicrobial peptides by means of action on the Toll pathway [17]. Prophenoloxidase activation is a kind of innate immune response in invertebrates. Once an insect is injured or infected, a pro-PO zymogen is activated by a certain protease. Phenoloxidase (PO) hydroxylates monophenols to *o*-diphenols and then oxidizes *o*-diphenols to quinones, which can polymerize to form melanin at the injury site or around invading organisms [18], [19]. Quinones are included in the production of cytotoxic molecules such as hydroxyl radicals and superoxides, and play an important role in killing the invading pathogens or parasites [18], [20]. However, over production of quinones could harm the host. Therefore the role of the serpins is to balance the production of quinones by regulating the pro-PO system. For example, serpin-2 from the African malaria mosquito, *Anopheles gambiae*, is a key regulator of the melanization response [21]. Serpin-1, -2, -3, -4, -5, -6, -7 have previously been identified and characterized from the tobacco hornworm *Manduca sexta*. All of them could inhibit and regulate proteases that lead to activation of the pro-PO system and the cytokine spatzle function in cascades [19], [22]–[30].

The diamondback moth, *Plutella xylostella* (Linn.), (Lepidoptera: Yponomeutidae), is a worldwide pest of cruciferous crops. Larvae of *P*. *xylostella* feed on the leaves of the cruciferous plants until harvesting and cause great economical loss in yield and quality of the crop. It has been estimated that around 1 billion US$ are spent annually on its control throughout the world [31], [32]. The application of chemical insecticides is the only effective control method for *P*. *xylostella*. However, due to extensive insecticide applications it has rapidly developed resistance [33], [34]. High insecticide tolerance in pests and environmental safety concerns can happen when outbreaks of the pest occur. These can threaten both human health and the economy [35]–[37], hence there is increasing interest in development of integrated pest management (IPM) strategies which encourage minimum use of chemical insecticides [38]. Biological control plays an important role in sustaining successful IPM, which is a main means of reducing pesticide residues and ensuring food safety. Bio-insecticides, which are important components of biological control, play an important part in IPM [39].

Destruxin A is one of the most potent mycotoxins of bio-insecticides, which can be synthesized by various species of entomopathogenic fungi such as *Metarhizium anisopliae*, *Aschersonia* sp, *Lecanicillium longisporum* and *Beauveria felina* during the infection process [40]–[42]. It plays critical roles in pathogenesis and has insecticidal activities to a wide range of insect pests [43]–[46]. Previous studies have shown that destruxin A could influence the Ca^2+^ channel in muscle cells, suppress the hydrolytic activity of V-type ATPase and inhibit the immune response [47]–[49]. However, the insecticidal mechanism of destruxin A has never been clearly studied in regards to its action on the innate immune response of insects.

In previous work, both digital gene expression (DGE) and two-dimensional electrophoresis (2-DE) approaches were adopted to examine the effects of destruxin A on the larvae of *P. xylostella* [50]. The results demonstrated that destruxin A influenced the expression of many serpins. However, the roles of the serpins in relation to the innate immune response in insects when challenged by destruxin A remained undetermined.

To gain a better understanding on the role of serpins, we first cloned insect serpin-4 and serpin-5 genes in *P. xylostella* and examined their related functional capabilities. The expression patterns of serpin-4, serpin-5 and another gene, serpin-2 whose full-length has already been submitted to GenBank (accession number AB282640), in different tissues and developmental stages of *P. xylostella* were analyzed by real-time fluorescence quantitative PCR and western blotting. RNAi-mediated knockdown of serpin genes was carried out by means of microinjection of a double-stranded RNA (dsRNA) to further investigate the consequences of these serpins. Our study was expected to provide new insight into the functional mechanism of destruxin A and contribute to the development of a new pest control approach.

## Results

### Molecular Characteristics of Serpins

**Figure 1:**
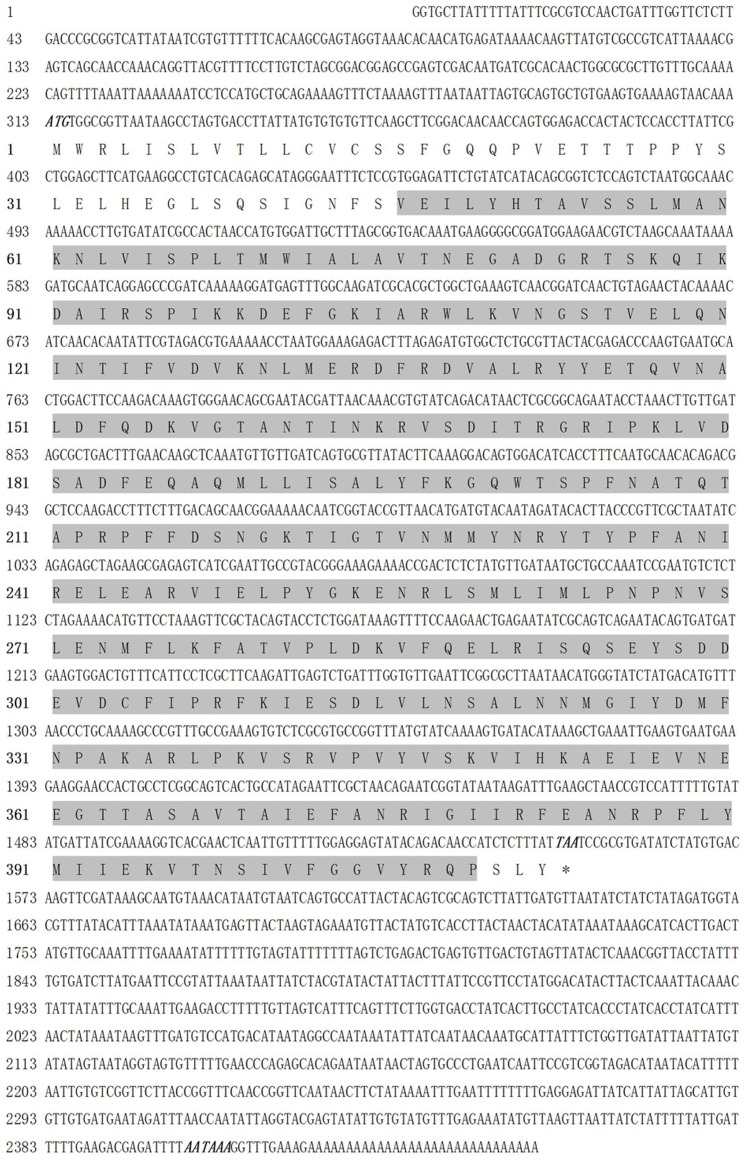
Nucleotide sequence (above) and deduced amino acid sequence (below) of the serpin-4 (GenBank accession No. KC686693). The serpin domain is shadowed. The asterisk (*) indicates the stop codon. Polyadenylation signal is bolded and italicized.

**Figure 2:**
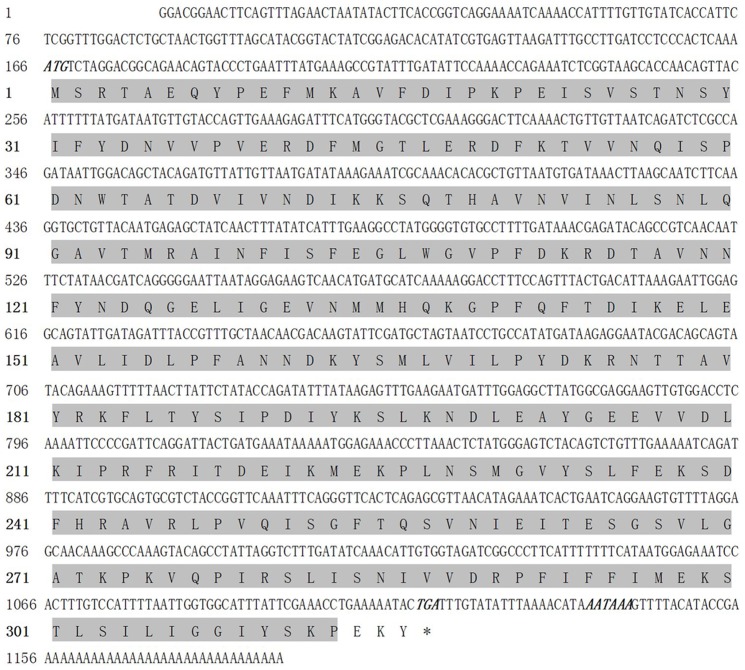
Nucleotide sequence (above) and deduced amino acid sequence (below) of the serpin-5 (GenBank accession No. KC505247). The serpin domain is shadowed. The asterisk (*) indicates the stop codon. Polyadenylation signal is bolded and italicized.

The full-length cDNA sequences of serpin-4 and serpin-5 were obtained by overlapping previous transcriptome data with the amplified fragments from the corresponding clone. The sequences were deposited in GenBank under accession number KC686693 (serpin-4) and KC505247 (serpin-5). The complete sequence of serpin-4 cDNA contained a 5′-untranslated region (UTR) of 312 bp, a 3′-UTR of 895 bp, and an open reading frame (ORF) of 1239 bp encoding a polypeptide of 412 amino acid residues (Fig. 1). The molecular weight (Mw) of serpin-4 was predicted to be 46.70 kDa and its isoelectric point (pI) was 7.63. The complete sequence of serpin-5 cDNA included an open reading frame of 951 bp flanked by a 5′- UTR of 165 bp and a 3′-UTR of 70 bp that encodes a polypeptide of 316 amino acids (Fig. 2). The Mw of serpin-5 was predicted to be 36.11 kDa and the pI was 5.43. SignalP software analysis showed that the deduced protein of serpin-4 contained a putative signal peptide of 18 amino acids, and no signal peptide for serpin-5. The domains of the serpin family were identified from serpin-4 (position 46–409 nt) and serpin-5 (position 1–313 nt) by SMART analysis. The E-value was 3.28e–70 and 1.63e–9 respectively.

### Phylogenetic Analysis of Serpins

**Figure 3:**
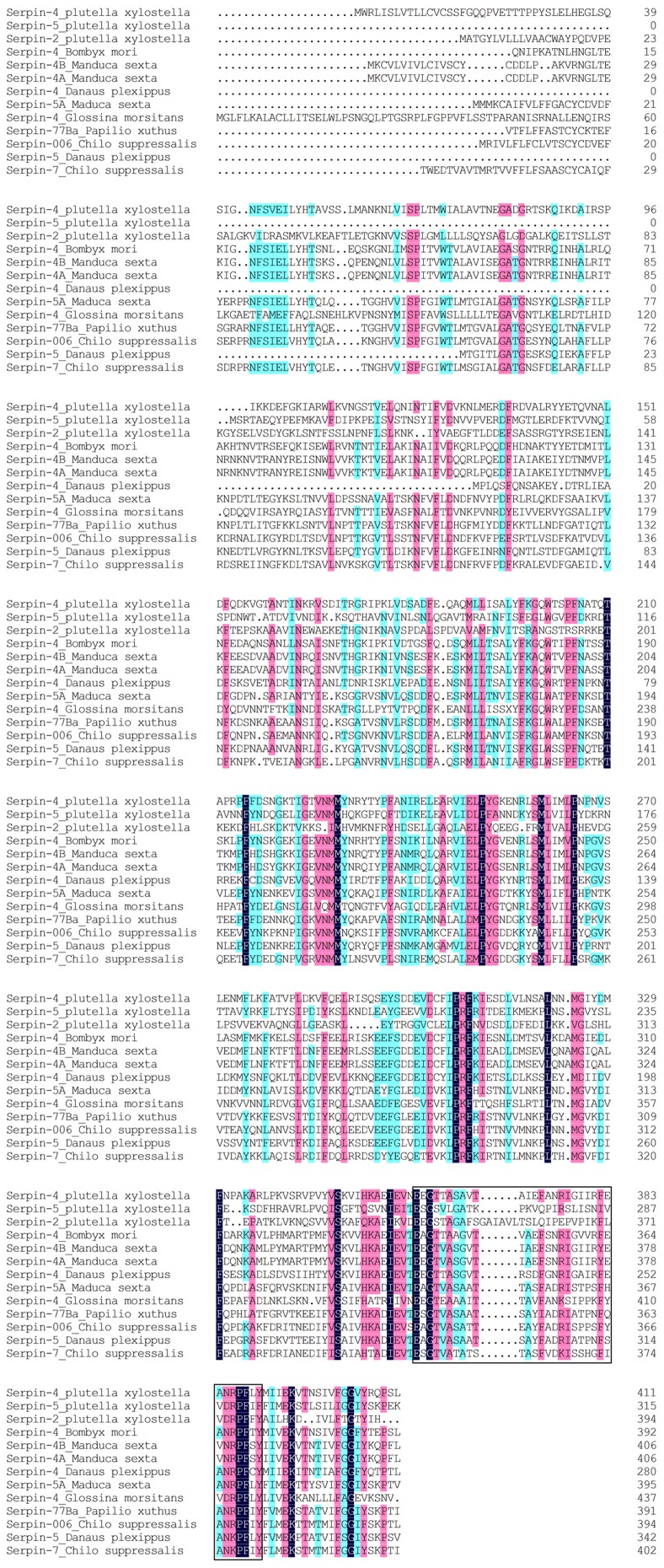
Multiple alignment of serpin-4 and serpin-5 with other known serpins. Here serpin-2 (*P. xylostella*, BAF36820), serpin-4 (*Bombyx mori*, ACZ81437), serpin-4B (*Manduca sexta*, AAS68504), serpin-4A (*Manduca sexta*, AAS68503), serpin-4 (*Danaus plexippus*, EHJ70588), serpin-5A (*Manduca sexta*, AAS68507), serpin-4 (*Glossina morsitans*, AFG28186), serpin-77Ba (*Papilio xuthus*, BAM10360), serpin-006 (*Chilo suppressalis*, AFQ01142), serpin-5 (*Danaus plexippus*, EHJ70286) and serpin-7 (*Chilo suppressalis*, AFQ01143) are included. The reactive centre loop regions are boxed.

**Figure 4:**
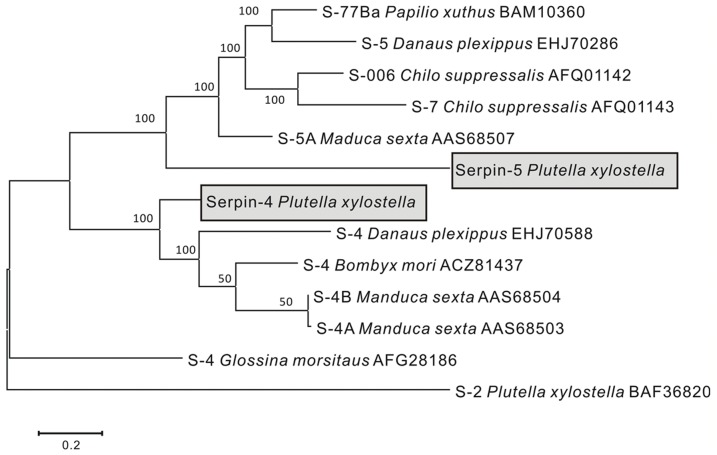
Consensus Neighbour-Joining tree based on the sequences of serpins. Maximum likelihood tree reconstruction based on sequences of serpins (length approximately 449) of representative sequences from Genbank under the WAG+G substitution model was carried out. The estimated value of the gamma shape parameter (+G) was 1.70. The bootstrap values are indicated. Serpins number (abbreviated as S in tree), accession number and species name are shown along the sequence in the tree. Sequences used in this study are highlighted in the tree via grey colour.

Amino acid sequences of selected serpins were aligned with the ClustalW2 programme embedded in the programme Mega 4. The deduced amino acid sequences of serpin-4 and serpin-5 shared homology with other known serpins of insects, such as, serpin-2 of *P. xylostella* (BAF36820), serpin-4 of *Bombyx mori* (ACZ81437) (Fig. 3). The multiple sequence alignments showed that several amino acid residues of serpins were conserved in different species (Fig. 3). The conserved regions of the reactive centre loop (RCL) were also identified in serpin-4 and serpin-5. A Neighbour-Joining (NJ) phylogenetic tree was constructed based on amino acid sequences of serpins using MEGA 4 programme (Fig. 4). Our serpin-4 of *P. xylostella* was clustered with serpin-4 of *Bombyx mori*, serpin-4B of *Manduca sexta*, serpin-4A of *Manduca sexta*, serpin-4 of *Danaus plexippus*, and serpin-5 was clustered with serpin-77Ba of *Apilio xuthus*, serpin-006 of *Chilo suppressalis*, serpin-5 of *Danaus plexippus* and serpin-7 of *Chilo suppressalis*. The identity of nucleotide sequences between serpin-2 and serpin-4, serpin-2 and serpin-5, serpin-4 and serpin-5 was 40.03%, 31.12%, 36.07% respectively, and that for protein sequences was 28%, 25% and 31%.

### The Distribution of Serpins in Different Tissues and Developmental Stages

**Figure 5:**
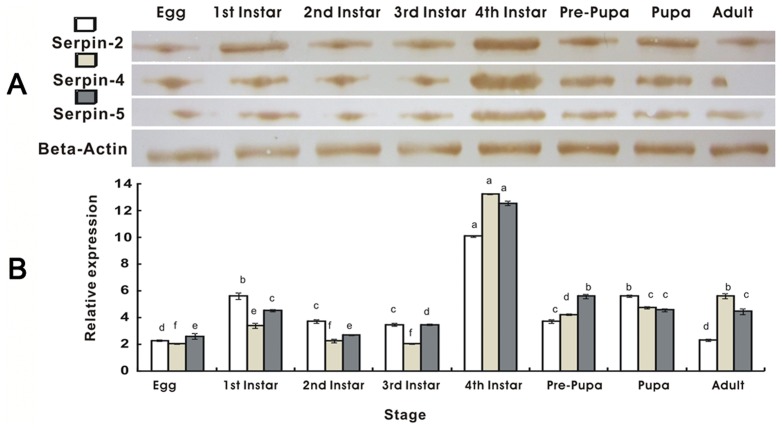
Expression of serpin-2, serpin-4 and serpin-5 in different lifestages of *Plutella xylostella* detected by Western Blot (A) and qRT-PCR (B). The mRNA and protein levels were normalized relative to the β-Actin. The different letters above the columns indicate significant differences in serpin gene expression during *P. xylostella* development (*P*<0.05). Each point represents mean value ± S.E.M of three independent experiments with three individuals in each replicate. Western Blot analysis visualized by DAB.

**Figure 6:**
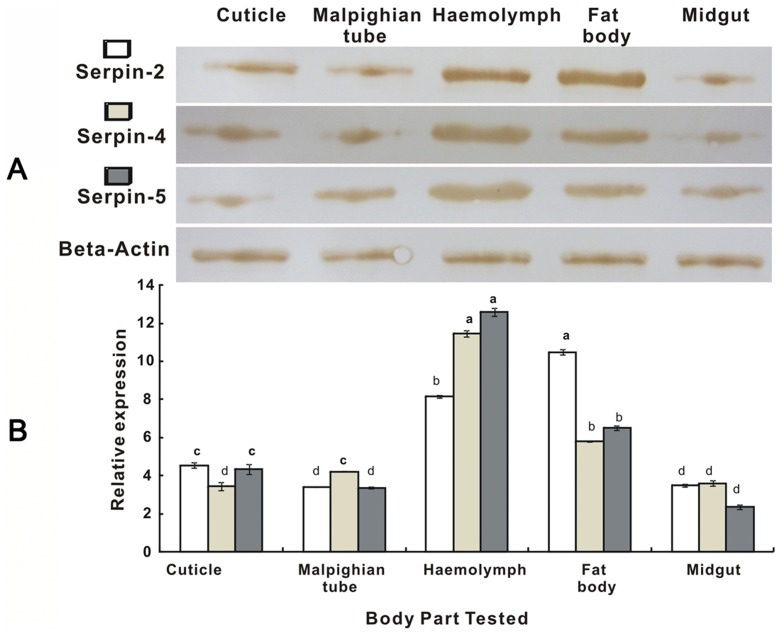
Expression of serpin-2, serpin-4 and serpin-5 in different tissues of *Plutella xylostella* detected by Western Blot (A) and qRT-PCR (B). The mRNA and protein levels were normalized relative to the β-Actin. The different letters above the columns indicate significant differences in serpins genes expression during *P. xylostella* development (*P*<0.05). Each point represents mean value ± S.E.M of three independent experiments with three individuals in each replicate. Western Blot analysis visualized by DAB.

The qRT-PCR and western blot were employed to investigate the expression of serpin-2, serpin-4 and serpin-5 genes at all developmental stages and within different tissues of the 4^th^ instar larvae of *P. xylostella*. RNA from eggs, larvae (1^st^ to 4^th^ stages), prepupae, pupae and adults were all normalized to β-actin. The mRNA transcripts and proteins of serpin-2, serpin-4 and serpin-5 could be detected in all the examined stages and tissues including the cuticle, midgut, fat body, Malpighian tubes and hemolymph. The results demonstrated that serpin-2, serpin-4 and serpin-5 were highly expressed in the 4^th^ instar larva. The expression levels of serpin-4 and serpin-5 were significantly increased in 4^th^ instar larvae compared with other stages (Fig. 5). Simultaneously, expression quantity of serpin-2, serpin-4 and serpin-5 in various tissues showed significant difference. The high levels of these serpins were detected in the fat body and heamolymph in contrast to the other tissues. The highest expression of serpin-2 was in the fat body while serpin-4 and serpin-5 was in the hemolymph (Fig. 6).

### Expression of Serpins after RNAi

**Figure 7:**
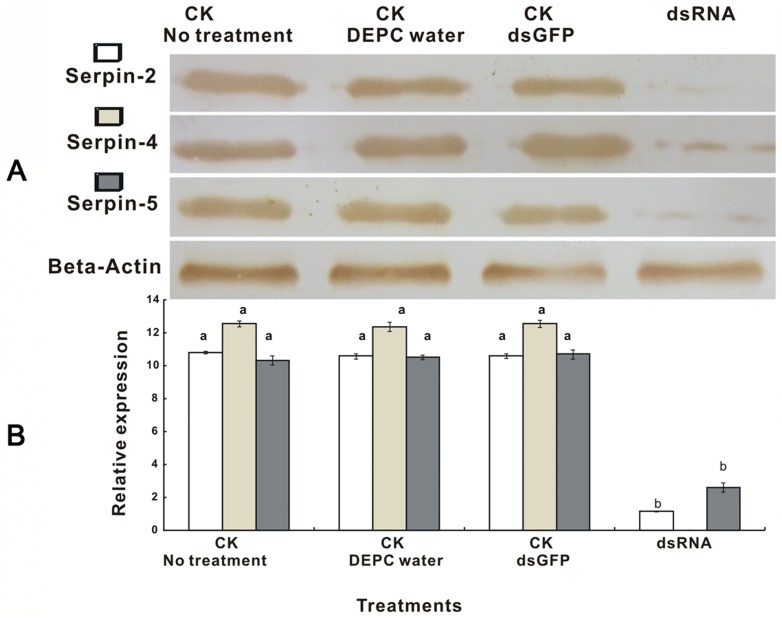
Detection of the efficiency of RNAi and impact on serpin-2, serpin-4 and serpin-5 mRNA levels by Western Blot (A) and qRT-PCR (B). The mRNA and protein levels were normalized relative to the β-Actin. The different letters above the columns indicate significant differences in serpins genes expression during *P. xylostella* development (*P*<0.05). Each point represents mean value ± S.E.M of three independent experiments with three individuals in each replicate. Western Blot analysis visualized by DAB.

**Figure 8:**
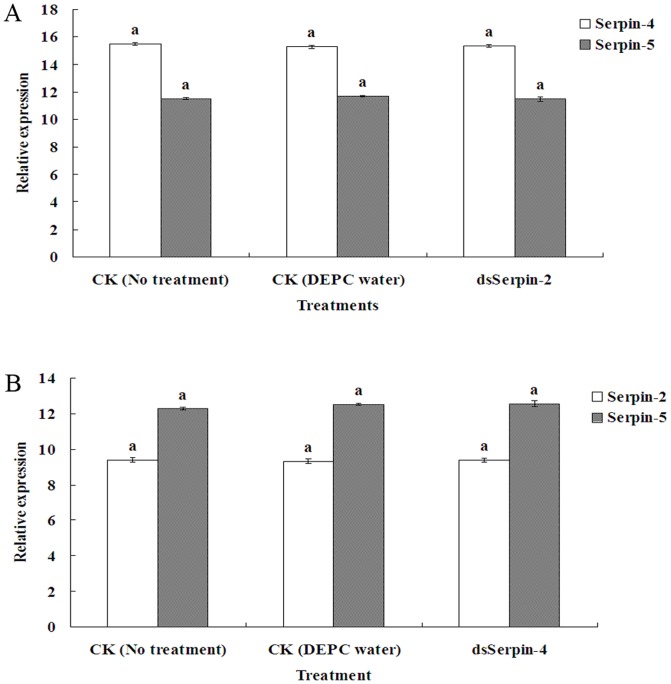
Expression of serpin-4 and serpin-5 after injecting dsSerpin-2 (A), expression of serpin-2 and serpin-5 after injecting dsSerpin-4 (B). Each value was shown as values ± S.E.M of three independent experiments. Significant differences were indicated with different letters at P<0.05.

The RNAi experiments were performed with injection of dsRNA that targeted special regions of the serpin genes. The mRNA relative expression levels and protein expression levels were detected by using qRT-PCR and Western bolt to investigate the RNAi-mediated knockdown efficiency of the serpin-2, serpin-4 and serpin-5 genes in 4^th^ larva of *P. xylostella*. The results revealed that the transcript levels of serpin-2, serpin-4 and serpin-5 were decreased 89.35%, 100% and 74.71% respectively compared to the three controls (no treatment, DEPC water, dsGFP) (Fig. 7). The Western blot analysis of efficiency for RNAi at protein levels confirmed the results of the transcript levels. These results demonstrated that it was a highly efficient RNAi-mediated knockdown of serpin-2, serpin-4 and serpin-5. The data showed that there weren't cross activity knockdown effects of dsSerpin-2 on serpin-4 and serpin-5, also dsSerpin-4 on serpin-2 and serpin-5 (Fig. 8).

### Analysis of effect following RNAi and Destruxin A Treatment

**Figure 9:**
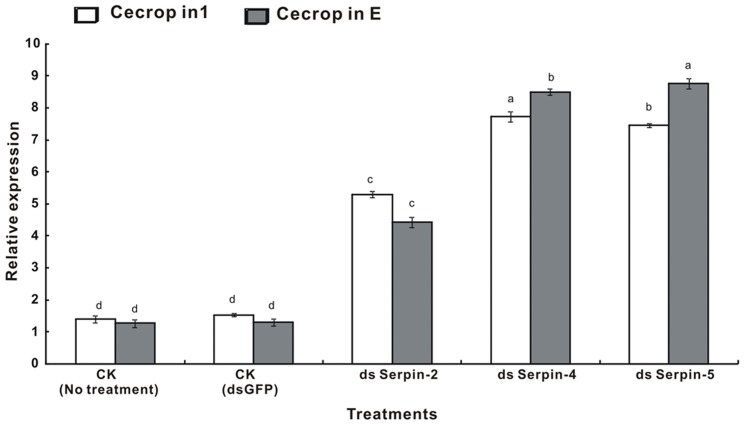
Expression of antimicrobial peptides (cecropin1 and cecropinE) after RNAi detected by SYBR Green real-time PCR. β-actin gene was used as an internal control to calibrate the cDNA template for all the sample. Each value was shown as values ± S.E.M of three independent experiments. Significant differences were indicated with different letters at *P*<0.05.

To reveal the effect of RNAi-mediated knockdown of serpin-2, serpin-4 and serpin-5 on the expression of antimicrobial peptides, the mRNA relative levels of cecropin1 and cecropinE were measured by qRT-PCR collected 24 h after microinjection of dsRNA. The transcript analysis revealed that the mRNA abundance of cecropin1 and cecropinE treated by dsSerpin-2, dsSerpin-4 and dsSerpin-5 increased significantly, compared with the two controls (no treatment and dsGFP). Moreover, the expression levels of the two antimicrobial peptides induced by dsSerpin-4 and dsSerpin-5 were significantly higher than dsSerpin-2 (Fig. 9).

**Figure 10:**
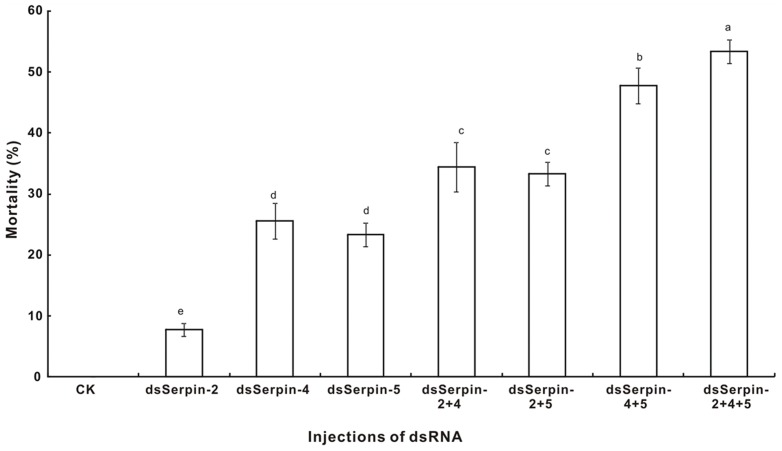
Effect of different serpin knockdown on larval mortality of *Plutella xylostella*. Each treatment was injected with 2 µl of a solution containing a total of 5 µg dsRNA. Percentage mortality 24 h after RNAi was calculated. Each value was shown as values ± S.E.M of three independent experiments. Significant differences were indicated with different letters at *P*<0.05.

When investigating the physiological effects of injecting different dsRNA and destruxin A on the body melanization and mortality of *P. xylostella*, results showed that no body melanization and mortality were observed in the control experiment, while different levels of body melanization and mortalities were noticed in the destruxin A and dsSerpin treatments. All dead insects were melanized over their entire bodies. The mortality of *P. xylostella* after injecting single dsRNA of dsSerpin-4, dsSerpin-5 and dsSerpin-2 were 25.56%, 23.33% and 7.78%, respectively. Meanwhile, the mortalities of *P. xylostella* after injecting dsRNA were: 34.44% with dsSerpin-2+dsSerpin-4, 33.33% with dsSerpin-2+dsSerpin-5, and 47.78% with dsSerpin-4+dsSerpin-5. Moreover, when injecting the three dsRNA's simultaneously at least 55.56% higher mortality was recorded compared to the mortalities obtained in either the single or the double injection experiments (Fig. 10). These results revealed that RNAi of serpins have a superimposed effect on the mortality of *P. xylostella* larvae, and that the superimposed effects of dsSerpin-4 and dsSerpin-5 were significantly higher than dsSerpin-2.

**Figure 11:**
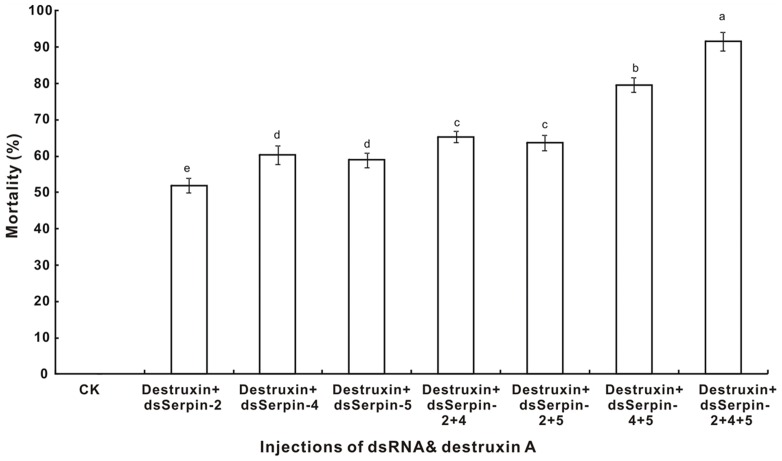
Insecticidal efficacy of destruxin A and serpin knockdown on larvae of *Plutella xylostella*. Each treatment was injected with 2 µl of a solution containing 200 µg/ml (LC_50_) destruxin A and a total of 5 µg dsRNA. The control treatment was injected with 2 µl of PBS buffer. Percentage mortality 24 h after treatment was calculated. Each value was shown as values ± S.E.M of three independent experiments. Significant differences were indicated with different letters at *P*<0.05.

**Figure 12:**
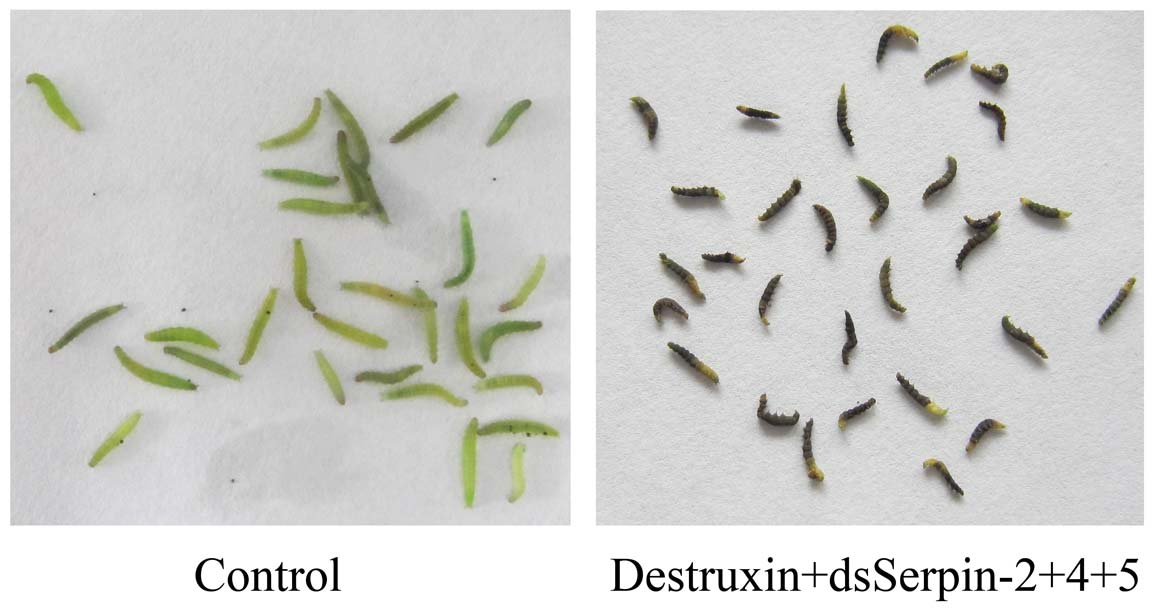
Melanization of *Plutella xylostella* treated with destruxin and dsSerpins. The treatment group was injected with 2 µl of a solution containing 200 µg/ml destruxin A and a total of 5 µg dsRNA. The control treatment was injected with 2 µl of PBS buffer.

When injection with destruxin A mixed with dsSerpins, our results showed that dsSerpins mixed with destruxinA could increase mortality of *P. xylostella*. The mortality of *P. xylostella* increased with the superposition of dsSerpins and destruxin A. For example, the mortality of *P. xylostella* was 60.33%, 79.67% and 91.67% in the destruxin A plus dsSerpin-4 group, the destruxin A plus dsSerpin-4 and dsSerpin-5 group, and the destruxin A plus dsSerpin-4, dsSerpin-5 and dsSerpin-2 group, respectively (Fig. 11). All dead insects were accompanied by melanization of the entire body (Fig. 12). Therefore, these results clearly demonstrated that RNAi-mediated knockdown of serpin genes increases mortality of *P. xylostella* larvae when challenged by destruxin A.

### The effect of serpin silencing on PO activity

**Figure 13:**
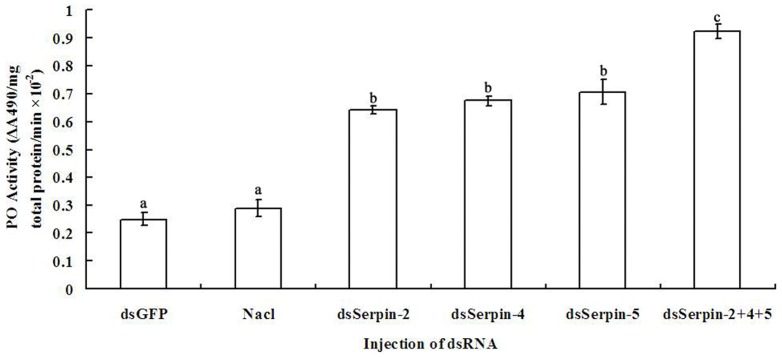
Hemolymph phenoloxidase (PO) activity in serpin silenced larvae of *Plutella xylostella*. Hemolymph was collected at 24(dsSerpin-2, dsSerpin-4 and dsSerpin-5). Larvae injected with dsGFP and saline buffer were used as control groups. The PO activity was measured using L-dopa and defined as ΔA_490_ per mg total protein. Each value was shown as values ± S.E.M of three independent experiments. Significant differences were indicated with different letters at *P*<0.05.

To determine whether the knockdown of the three serpin genes would cause an increase in PO activity, hemolymph was extracted from the serpin silenced larvae of *P. xylostella* and subjected to a total PO enzymic activity assay. The results showed that knockdown of serpin-2, -4, -5 significantly increased the total PO activity by 2.56, 2.68 and 2.84 fold respectively compared to the control experiment. When co-silencing of serpin-2, -4, and -5, it increased the PO activity by 3.68 fold than that of non-serpin silencing experiments. On the contrary, there was no significant change of PO activity in dsGFP treatment group (Fig. 13). Clearly the total PO activity is linearly related to the knockdown in serpin-2, -4 and -5; suggesting cooperation between the three gene products.

## Discussion

In our previous digital gene expression and two-dimensional electrophoresis study, we found that destruxin A deeply influenced the expression of the serpin family genes including serpin-2, serpin-4 and serpin-5 [50]. In the current study, these serpins of *P. xylostella* when identified and characterized, showed high similarity with the known serpin family with high conservation of the reactive centre loop (RCL) (Fig. 3). All these sequence features suggest that serpin-4 and serpin-5 are typical serpins from *P. xylostella*.

Several serpins play important roles in the innate immunity of insects. They are involved in many immune processes, such as body melanization, blood coagulation, encapsulation complement activation and synthesis of antimicrobial peptides [15]. As the unique defense system of invertebrates, it is the prophenoloxidase (proPO) system that controls the body melanization process, playing an important role in the insect immune response. Recently, almost all known members of serpins that regulate the proPO system were identified from the tobacco hornworm *Manduca sexta* [19], [22]–[30]. Among them, serpin-4 and serpin-5 have been proven to inhibit the prophenol oxidase activity in the innate immune responses of *M. sexta* [19]. Serpin-2 has been identified as a key regulator of the body melanization response in the African malaria mosquito *Anopheles gambiae* [21]. It is suggested that all three of these serpin genes are related to the body melanization process that is induced by the proPO system in *P. xylostella*.

The information on the spatial and temporal distribution of serpins to insect immunity might provide useful cues to understanding the biological role or physiological function of serpins. In the current study, we first investigated the differential expression of serpins in different tissues and life-stages when there was no challenge by invaders. Serpin-2, serpin-4 and serpin-5 mRNA transcripts could be detected in all examined stages and tissues, including the cuticle, midgut, fat body, Malpighian tubes and the hemolymph. The results demonstrated the highest expression levels of serpin-2, serpin-4 and serpin-5 appeared at the 4^th^ larval stage (Fig. 5). Abundant transcripts of serpin-2, serpin-4 and serpin-5 were detected in both the fat body and hemolymph (Fig. 6). Previous studies have shown that serpin-4 and serpin-5 mRNAs in *Manduca sexta* are generally expressed at a low level in both larval hemocytes and fat body and that they increase dramatically once a bacterial challenge is encountered. Serpin-2 is an intracellular protein and is expressed in the cytoplasm of hemocytes after bacterial challenge [19], [51]. Serpin-3 is present at a low level in larvae and increases dramatically in concentration after microbial challenge while serpin-1 mRNA is constitutively expressed in the larval fat body [52]. In the current study, it may be because the fat body and hemolymph are important immune organs taking part in body melanization of the insect innate immunity, that the genes related to immune response, including serpins maintain a higher level of expression in these two tissues than in the others. In the current study, the expression of serpin-2, serpin-4 and serpin-5 were highest at the 4^th^ larva stage of *P. xylostella*, this may be because 4^th^ instars are getting ready to pupate, a time when melanization is critical for cuticular hardening.

To elucidate the functions of serpins, the method used in other studies was isolating and purifying serpins and then injecting them into insects [19], [24], [30]. RNAi has been widely used to investigate the functions of a number of genes in different insects of Lepidoptera [53]. In *P. xylostella*, RNAi silencing has been previously used to investigate the role of a cytochrome P450, CYP6BG1 in the 4^th^ larval stage resistance to the pesticide permethrin [54]. This is also the first time that we used this method to study the functions of serpins. The RNAi-mediated knockdown of these serpins in the current study was performed by injecting a specific dsRNA into 4^th^ instar larvae, and while the expression of serpin-2, serpin-4 and serpin-5 after RNAi treatment for 24 h decreased dramatically, the mortality caused by dsSerpin increased compared to the control (Fig. 7&9). Our findings are similar to the study of Jiang et al. [55] in which the survival rate of *Tenebrio molitor* improved by injection of three serpins. Also, the expressions of two antimicrobial peptides including cecropin1 and cecropinE in our current study increased significantly (Fig. 9). These results were in agreement with previous observations in serpin43Ac-deficient *Drosophila* concerning the expression of antimicrobial peptides induced by the Toll pathway [17], in which the study suggested serpin-2, serpin-4 and serpin-5 might play an important role in regulation of the Toll signal pathway that manipulates the expression of antimicrobial peptides.

Michel et al. [56] reported that knockdown of serpin-2 can induce significantly shorter longevity in the adult mosquito *Anopheles gambiae*. However, our study has demonstrated that the RNAi of serpins have a superimposed effect on mortality of *P. xylostella* larvae (Fig. 10), and the insecticidal efficacy of dsSerpin-4 and dsSerpin-5 was obviously better than dsSerpin-2. After injecting dsSerpin, black necrotic spots were noted on larvae of *P. xylostella* with all dead insects being accompanied by body melanization (Fig. 12). This indicated that either serpin-2, serpin-4 or serpin-5 in *P. xylostella* might act as one of the vital regulators of proPO activities via inactivating prophenoloxidase-activating proteases. These results are consistent with previous reports about flies when deficient of serpin27A exhibiting the spontaneous melanization in larvae and adults of *Drosophila* [57]. They are also similar to functions of serpins recorded in *Manduca sexta* [19].

In testing the insecticidal bioassay of destruxin A to *P. xylostella* accompanied by RNAi-mediated knockdown of serpin-2, serpin-4 and serpin-5, we found that the silencing of these three serpin genes could significantly increase the mortality of *P. xylostella* larvae challenged by destruxin A (Fig. 11). Similar results were also found in the study of Rodriguez-Cabrera et al. [58], which showed the RNAi of a trypsin-like serine-protease gene reduced the susceptibility of *Spodoptera frugiperda* to a *Bacillus thuringiensis* protoxin. In *Manduca sexta*, serpins have been proven to inhibit the PO activity in a dose-dependent manner [19], [59], however, in our findings, RNAi-mediated knockdown of serpins significantly increased the total PO activity measured by using dopamine as a substrate in the larvae of *P. xylostella* (Fig. 13). This might be because of the high efficiency and endurance of RNAi, PO activity was enhanced continuously due to the lack of serpins.

For destruxins to be considered as important bio-insecticides, their functional mechanisms must be clarified. Destruxins give rise to a wide range of biological actions in insects, including: induction of membrane depolarization in skeletal muscle [48], inhibition of fluid secretion rate by the Malpighian tubules [60], inhibition of the hydrolytic activity of V-type ATPase of brush border membrane vesicles [47], induction of disruption of the epithelial cell and membrane oxidative stress in cells [61], suppression of the immune response [49], and destruxin A can also inhibit the expression of various antimicrobial peptides produced by the innate immune system in insects [49]. However, the mechanism of destruxin is still unclear. In our previous study, we detected the up-regulated trend of serpins after treatment of destruxin A in a live insect, the expression of serpins should be down-regulated in the dead insect, so in the current experiment, we found depletion of serpins increased insecticidal efficacy of destruxin A. Therefore, there is an interaction between destruxin A and the insect. A possible reason for the killing of the insect due to destruxin is that destruxin inhibits serpin genes. We deduced the possibility that the functional mechanism is that destruxin A suppress the expression of serpins that regulate the body melanization. Excessive melanization usually occurrs due to the absence of serpins, and this induces the mass production of quinones, which are very toxic to most organisms. Certainly, the action mechanism of destruxin A is the result of many aspects, and further research is needed to further investigate this matter.

In conclusion, our study presented a preliminary molecular characterization of the serpin genes in *P. xylostella* and an analysis of their expression patterns and function. The results demonstrate that the decrease in serpin-2, serpin-4 and serpin-5 expression levels leads to proPO-activation in the larvae of *P. xylostella* and therefore increases mortality of the insect. We deduced the possible function mechanism of destruxin A was that destruxin A inhibited the expression of serpin genes, then excessive melanization took place for lack of serpins regulation, which induced over production of quinones that harm and accelerate death of the host.

Our findings are expected to enhance the understanding of the potential insecticidal mechanism of destruxin A, and constitute a well-defined potential molecular target for novel insecticides in the future.

## Material and Methods

### Insect rearing and destruxin A preparation

The strain of susceptible *P. xylostella* was reared in the Engineering Research Centre of Biological Control, Ministry of Education, South China Agricultural University (SCAU), and was maintained for 10 generations without exposure to insecticides. Rearing conditions were set at 25±1°C, 65% RH, a 14-h light/10-h dark photoperiod and 1000-1500 lx intensity. Destruxin A was isolated and purified from strain MaQ-10 of *Metarhizium anisopliae* in the laboratory [62]. The purity of destruxin A was analyzed by high performance liquid chromatography (HPLC). It was then diluted with phosphate buffered saline (PBS, PH7.4).

### RNA extraction and cDNA cloning

Total RNA was isolated from the 4^th^ larval instar stage of *P. xylostella* using the Total RNA Kit according to the manufacturer's specifications (Omega, USA). First-strand cDNA was synthesized with PrimeScript 1st strand cDNA Synthesis Kit (TaKaRa, Japan). Briefly, 1 µg of total RNA, 1 µL of Oligo dT primer (50 µM), 1 µL of dNTP Mixture (10 mM) and the RNase free deionized water was added up to 10 µL, kept for 5 min at 65°C and then immediately cooled on ice. Then 4 µL of 5× PrimeScript buffer, 0.5 µL of RNase Inhibitor, 1 µL of PrimeScript RTase and RNase free deionized water was added to make up the final volume to 20 µL. The reaction mixture was incubated under the conditions of 42°C for 60 min, followed by 70°C for 15 min and then cooled on ice. Two pairs of primers were designed based on the partial cDNAs for serpin-4 and serpin-5 obtained from our previous transcriptome sequences of susceptible *P. xylostella* and the first-strand cDNA (1 µl) was used as a template for the PCR reaction mixture containing 0.5 mM of each primer, 0.1 mM dNTP and 1.0 U of HiFi-Taq DNA polymerase (GenStar, Beijing, China) in a total volume of 25 µl. The PCR program was set up as following: initial preheating step for 5 min at 94°C, followed by 35 cycles of 94°C for 30 s, 46°C for 30 s, 72°C for 1 min, with the final extension step at 72°C for 10 min with the primer pairs *Px*Sp4F, *Px*Sp4R and *Px*Sp5F, *Px*Sp5R (Table 1). The amplified products were recovered in a 1% agarose gel and purified by using the Gel Extraction Kit (Omega, USA), cloned into the pMD18-T vector (TaKaRa, Japan) in *E. coli*. The sequencing reaction was performed by the Beijing Genomic Institute (Beijing, China). To obtain the full-length cDNA, the RACE Kit (Clontech, Japan) was used. Specific primers for the 5′- and 3′- Rapid Amplification of cDNA Ends (RACE) were designed based on previous sequencing results. The nested primers 5*Px*Sp4a, 5*Px*Sp4b and 5*Px*Sp5a, 5*Px*Sp5b were used for 5′-RACE; 3*Px*Sp4a, 3*Px*Sp4b and 3*Px*Sp5a, 3*Px*Sp5b were used for 3′-RACE (Table 1). Using the 5′- and 3′-RACE cDNA as templates, PCR was performed using the 5*Px*Sp4a, 5*Px*Sp5a, 3*Px*Sp4a, 3*Px*Sp5a and Universal Primer Mix (UPM, Clontech, Japan) under the following conditions: 5 min at 94°C, 35 cycles of 30 s at 94°C, 30 s at 55°C and 2 min at 72°C, and then finally 10 min at 72°C. Nested PCR was performed with the first PCR products as templates using the Nested Universal Primer A (NUP, Clontech, Japan) and 5*Px*Sp4b, 5*Px*Sp5b, 3*Px*Sp4b, 3*Px*Sp5b. The nested PCR was carried out under the same reaction conditions as the first PCR. The PCR products were also cloned into pMD18-T vector for sequencing.

**Table 1:** The primer sequences used in the study.

Primers	Forward	Reserve
*Px*Sp4	AAGGGGCGGATGGAAGAACGTCT	GTCTTGGAGCCGTCTGTGTTGCA
*Px*Sp5	GGACGGAACTTCAGTTTAGA	CTGTTGGTGCTTACCGAGAT
5*Px*Sp4a	GATGTGGTACGTTGGCATCTCGTT	
5*Px*Sp4b	GCAAACGCCTCAGCATTTCCAGTT	
5*Px*Sp5a	AAGTCCCTTTCGAGCGTACCCATGA	
5*Px*Sp5b	AATTCAGGGTACTGTTCTGCCGTCC	
3*Px*Sp4a	ATTGGAACTGGAAATGCTGAGGCG	
3*Px*Sp4b	ACGACGAAACAAAACGAATGGACC	
3*Px*Sp5a	CACCATTCTCGGTTTGGACTCTGC	
3*Px*Sp5b	GATTTGCCTTGATCCTCCCACTCA	
*Px*Sp4Or	CCGGGATCCATGTGGCGGTTAATAAGCCTAGT	CGCGCGGCCGCTTAATAAAGAGATGGTTGTCTGT
*Px*Sp5Or	CCGGGATCCATGTCTAGGACGGCAGAACAGT	CGCGCGGCCGCTCAGTATTTTTCAGGTTTCGAA
*Px*Sp2Or	CCGGGATCCATGGCGACTGGATATTTAGTTCT	CGCGCGGCCGCTTAGTGAATGTAAGTGCCAGTAA
*Px*Sp2RT	ACCCCAACTTCCTGTCGCT	GGTTTCCTTCTCTGCCCAT
*Px*Sp4RT	GAGACCACTACTCCACCTT	TTTGTTTGCCATTAGACTG
*Px*Sp5RT	GAGCAACAAAGCCCAAA	CGAATAAATGCCACCAA
Actin	TGGCACCACACCTTCTAC	CATGATCTGGGTCATCTTTT
T7*Px*Sp2	GGATCCTAATACGACTCACTATAGGAGAGGCACCTACAGGAGC	GATGTCAAAACGGCAATC
T7*Px*Sp2	AGAGGCACCTACAGGAGC	GGATCCTAATACGACTCACTATAGGGATGTCAAAACGGCAATC
T7*Px*Sp4	GGATCCTAATACGACTCACTATAGGGATTGCTTTAGCGGTGA	AGTCGGTTTTCTTTCCC
T7*Px*Sp4	GATTGCTTTAGCGGTGA	GGATCCTAATACGACTCACTATAGGAGTCGGTTTTCTTTCCC
T7*Px*Sp5	GGATCCTAATACGACTCACTATAGGAGAAATCGCAAACACAC	ATGAAGAAAATGAAGGG
T7*Px*Sp5	AGAAATCGCAAACACAC	GGATCCTAATACGACTCACTATAGGATGAAGAAAATGAAGGG
T7GFP	GGATCCTAATACGACTCACTATAGGAAGGGCGAGGAGCTGTTCACCG	CAGCAGGACCATGTGATCGCGC
T7GFP	AAGGGCGAGGAGCTGTTCACCG	GGATCCTAATACGACTCACTATAGGCAGCAGGACCATGTGATCGCGC
Cecropin E	GGAATAAAAGATTCCAATTTCAA	CATCACGGATGTGCTGTCCCACT
Cecropin 1	GTCGCTGTCATCGGACAAGCCAC	TATACATTATTTAACCCGTAAAT

### Multiple sequences analysis

The sequences of the serpin-4 and serpin-5 cDNA were compared with other serpin sequences known within the Blast programme available on the National Center for Biotechnology Information (NCBI) website (http://www.ncbi.nlm.nih.gov/BLAST/). The deduced amino acid sequences of serpin-4 and serpin-5 were aligned by using the ClustalW2 software package (http://www.ebi.ac.uk/clustalw/index.html) [63]. SignalP was utilized to predict the signal peptide (http://www.cbs.dtu.dk/services/SignalP/). The protein motif features were predicted by Simple Modular Architecture Research Tool (http://smart.emblheidelberg.de/). A multi-species phylogenetic tree based on the amino acid sequences of serpins was constructed with MEGA4.0 software using the neighbour-joining method and 1000 bootstrap replicates [64].

### Expression of recombinants and polyclonal antibodies production

The full length of the open reading frame (ORF) of serpin-4 cDNA sequence (313-1,551 bp), serpin-5 cDNA sequence (166-1,116 bp) and the previously reported serpin-2 (1,185 bp) gene in Genebank (gi|117970183|) were amplified with primers *Px*Sp4OrF, *Px*Sp4OrR, *Px*Sp5OrF, *Px*Sp5OrR, *Px*Sp2OrF and *Px*Sp2OrR (Table 1), among which the forward and reverse primers contain restriction sites *Bam H*I and *Not* I respectively. The amplification conditions of the first PCR run were denaturing at 94°C for 5 min, then 35 cycles of 94°C for 30 s, annealing at 46°C for 30 s, extension at 72°C for 2 min, and a final extension at 72°C for 10 min. The PCR products were digested with *Bam H*I and *Not* I (Thermo Scientific, USA) subcloned into pET-32a (+) vector. This plasmid construction was used for protein expression in *E. coli* (BL21) competent cells. The *E. coli* (BL21) was disrupted by an ultrasonic wave and solubilized in equilibrium buffer: 8 M urea, 0.1 M NaH_2_PO_4_, 0.01 M Tris base, pH 8.0, then purified with the Ni-NTA column (TransGen Biotech, China). Purified recombinant serpin proteins were used to immunize rabbits using a previously described method [65].

### Temporal and Spatial Expression of Serpins

**Figure 14:**
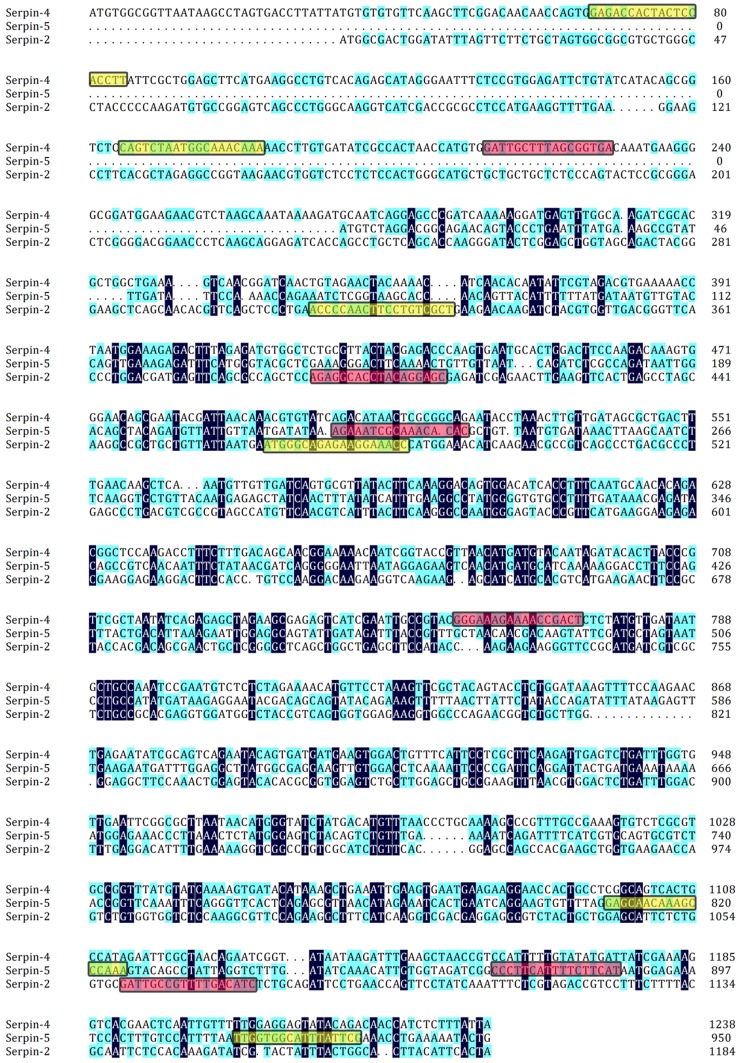
Nucleotide sequences alignment of open reading frame of serpin-2, serpin-4 and serpin-5 marked with the position of dsRNA and qPCR primers. The dsRNA primers are boxed in red, qPCR primers are boxed in yellow.

The temporal and spatial expression of serpin-2, serpin-4 and serpin-5 were further investigated by quantitative real-time PCR (qRT-PCR) and Western blot technique. Total RNA was isolated from *P. xylostella* at developmental stages containing egg, 1^st^, 2^nd^, 3^rd^ and 4^th^ instar larvae, prepupae, pupae, adults, and tissues including cuticle, hemolymph, fat body, midgut and Malpighian tubules. All samples were used for reverse transcription to obtain the first-strand cDNA as previously described. The qRT-PCR was performed using a BIO-Rad CFX-96 Real-Time PCR system with the iTaq Universal SYBR Green Supermix Kit (BIO-Rad, USA) using gene specific primers: *Px*Sp2RTF, *Px*Sp2RTR, *Px*Sp4RTF, *Px*Sp4RTR, *Px*Sp5RTF and *Px*Sp5RTR (Table 1). The position of qPCR primers in nucleotide sequences was shown in Fig. 14. As an endogenous control to normalize the expression levels with that average threshold cycle (Ct), a partial fragment of the *P. xylostella* β-actin gene (DQ494753) was amplified with ActinF and ActinR primers (Table 1). QRT-PCR was performed at 95°C for 3 min, followed by 39 cycles at 95°C for 10 s, 58°C for 30 s, and 72°C for 30 s, plus a final extension step at 72°C for 1 min. Each reaction was run in triplicate and the relative expression of genes was calculated using the (2^-△△Ct^) method. Western-blot analysis and protein extraction were performed according to methods previously described [66], [67]. In brief, proteins of various stages and tissues were extracted from *P*. *xylostella*. The protein concentration was quantified according to the Bradford method [68]. In total, 350 mg proteins were separated on a 12% SDS-PAGE gel, which was semi-dry transferred at 15 V for 25 min to 0.45 mm PVDF membrane (Bio-Rad, USA), immunoblotted with anti-*Px*Serpins serum (diluted 1∶5000) and anti-β-Actin serum (Cwbiotech, China). The IgG goat anti-rabbit antibody conjugated with HRP (BOSTER, China) was used as a secondary antibody (diluted 1∶3000), with a DAB Kit (BOSTER, China) used for the visualization of the protein band.

### RNA Interference (RNAi) and Destruxin A Treatments

For silencing of serpin-2, serpin-4 and serpin-5 in *P*. *xylostella*, double-stranded RNA (dsRNA) was synthesized by using T7 RNA polymerase (T7 RiboMAX Express RNAi system kit) (Promega, USA). According to the manufacturer recommendations, six pairs of primers (T7*Px*Sp2F and *Px*Sp2R, *Px*Sp2F and T7*Px*Sp2R, T7*Px*Sp4F and *Px*Sp4R, *Px*Sp4F and T7*Px*Sp4R, T7*Px*Sp5F and *Px*Sp5R, *Px*Sp5F and T7*Px*Sp5R) (Table 1) were designed to synthesize cDNA fragments of serpin-2 (391–1076 bp of ORF), serpin-4 (213–773 bp of ORF) and serpin-5 (221–887 bp of ORF), which contained the T7 promoter region in both sense and antisense strands. The position of dsRNA primers in nucleotide sequences was shown in Fig. 14. The recombinant plasmids were used as a template with a reactions protocol including preheating 95°C for 4 min, followed by 35 cycles of 95°C for 30 s, 48°C for 30 s and 72°C for 1 min, and a final extension step of 72°C for 10 min. The sequence was confirmed by sequencing (Beijing Genomic Institute, China). The Green Fluorescent Protein (GFP) (ACY56286) gene was used as a negative control. The primers GFPF and GFPR were used to amplify the GFP cDNA fragment (657 bp) (Table 1), and the dsGFP was also synthesized by using T7 RiboMAX™ Express RNAi System. The dsRNA was further purified following precipitation using the G25 micro spin column following the manufacturer's instructions (Amersham Biosciences, USA). The final dsRNA was dissolved in nuclease-free water, quantified by the absorbance at a wavelength of 260 nanometers (One A260 unit equals ∼40 µg/ml of dsRNA) and stored at −70°C.

On the first day corresponding to the 4^th^ instar stage, the susceptible larvae of *P. xylostella* were injected in the abdomen with 2 µl solution containing 5 µg dsRNA by using the microINJECTOR System (Tritech Research, USA). In addition, three controls were arranged: a positive control (injection of an equivalent volume of nuclease-free water), a negative control (injection of an equivalent volume of dsGFP), and an additional negative control of no treatment. In the target gene detection experiment, each group consisted of 30 individuals each with three replicates. Ten larvae were randomly selected at 24 h after the injection for mRNA and protein level detection respectively. To verify the effectiveness of RNAi, follow-on experiments including qRT-PCR and Western blot were performed as previously described. In order to eliminate the possibility that there are cross activity knockdown effects of dsSerpin-2 on serpin-4 and serpin-5, also dsSerpin-4 on serpin-2 and serpin-5, the expression levels of serpin-4 and serpin-5 were detected after injecting 2 µl solution containing 5 µg of dsSerpin-2, the expression levels of serpin-2 and serpin-5 were analyzed when injected with dsSerpin-4, the controls were set as above described. The cDNA for detecting effectiveness of RNAi was also used in detecting the expression of antibacterial peptides. Two pairs of primers for antibacterial peptides were designed (Cecropin1F and Cecropin1R, CecropinEF and CecropinER) (Table 1) for qRT-PCR.

In our previous study, the LC_50_ for 4^th^ instar larvae treated with destruxin A after 24 h was 200 µg/ml [50]. For the bioassay of *P. xylostella* treated with destruxin A and dsRNA, fifteen treatment groups were performed with each group comprising 30 individual larvae with three replicates. These groups were as follows: (1) Injection of 2 µl PBS buffer as the control; (2) injection of 2 µl solution containing 5 µg of single dsRNA (dsSp2, dsSp4, dsSp5) (three groups); (3) injection of 2 µl solution containing a total of 5 µg of two kinds of dsRNA (dsSp2+dsSp4, dsSp2+dsSp5, dsSp4+dsSp5) (three groups); (4) injection of 2 µl solution containing a total of 5 µg of all dsRNA (dsSp2+dsSp4+dsSp5); (5) injection of 2 µl solution containing 200 µg/ml destruxin A and 5 µg of single dsRNA; (6) injection of 2 µl solution containing 200 µg/ml destruxin A and 5 µg of two kinds of dsRNA; (7) injection of 2 µl solution containing 200 µg/ml destruxin A and 5 µg of all dsRNA. Injections were made into the abdomen region of the body with the injection point sealed immediately with wax. The body melanization process after injection for 24 h was examined and mortality was calculated to assess the efficiency of treatments with RNAi and destruxin A.

### Assay of Hemolymph Phenoloxidase (PO) Activity

The phenoloxidase assay was modified according to a previous method [69], [70]. Hemolymph was extracted from the experimental 4^th^ larval stage at 24 h after the dsRNA injection, with the supernate being used for the phenoloxidase assay. Protein concentration was measured using a Bradford protein assay kit (Bio-Rad, USA), hemolymph PO activity was detected using L-3, 4-dihydroxyphenylalanine (L-dopa) dissolved in water. Briefly, 2 mg of total hemolymph proteins in 435 µL of Tris-HCl (10 mM, pH 8.0) were mixed with 65 µl of freshly prepared L-dopa (3 mg/ml). After a 30 min incubation period at room temperature, 500 ml of 10% (v/v) acetic acid was added to the mixtures and PO activity was measured by monitoring the absorbance at 490 nm in a microplate reader (Bio-Rad, USA). PO activity was recorded as ΔA_490_ per mg total protein/min. Control samples were prepared using saline buffer instead of larvae hemolymph.

### Statistical Analysis

The relative expression of serpin genes was calculated using the CFX96 Real-Time system (Bio-Rad, USA). All data were expressed as the means (±SE) of three independent experiments. Statistical calculations were performed using SAS V9.0 statistical software. Significant differences were determined by using Duncan's multiple range test (DMRT) at the 95% confidence level (p<0.05).
